# Effect of Dysferlin Deficiency on Atherosclerosis and Plasma Lipoprotein Composition Under Normal and Hyperlipidemic Conditions

**DOI:** 10.3389/fphys.2021.675322

**Published:** 2021-07-22

**Authors:** Zoe White, Nadia Milad, Stephanie L. Sellers, Pascal Bernatchez

**Affiliations:** ^1^Department of Anesthesiology, Pharmacology, and Therapeutics, The University of British Columbia, Vancouver, BC, Canada; ^2^UBC Centre for Heart Lung Innovation, St. Paul’s Hospital, Vancouver, BC, Canada

**Keywords:** dysferlin, endothelial function, hyperlipidemia, lipids, atherosclerosis, plaque, vascular homeostasis

## Abstract

Dysferlinopathies are a group of muscle disorders caused by mutations to dysferlin, a transmembrane protein involved in membrane patching events following physical damage to skeletal myofibers. We documented dysferlin expression in vascular tissues including non-muscle endothelial cells, suggesting that blood vessels may have an endogenous repair system that helps promote vascular homeostasis. To test this hypothesis, we generated dysferlin-null mice lacking apolipoprotein E (ApoE), a common model of atherosclerosis, dyslipidemia and endothelial injury when stressed with a high fat, and cholesterol-rich diet. Despite high dysferlin expression in mouse and human atheromatous plaques, loss of dysferlin did not affect atherosclerotic burden as measured in the aortic root, arch, thoracic, and abdominal aortic regions. Interestingly, we observed that dysferlin-null mice exhibit lower plasma high-density lipoprotein cholesterol (HDL-C) levels than their WT controls at all measured stages of the disease process. Western blotting revealed abundant dysferlin expression in protein extracts from mouse livers, the main regulator of plasma lipoprotein levels. Despite abnormal lipoprotein levels, Dysf/ApoE double knockout mice responded to cholesterol absorption blockade with lower total cholesterol and blunted atherosclerosis. Our study suggests that dysferlin does not protect against atherosclerosis or participate in cholesterol absorption blockade but regulates basal plasma lipoprotein composition. Dysferlinopathic patients may be dyslipidemic without greater atherosclerotic burden while remaining responsive to cholesterol absorption blockade.

## Introduction

Dysferlin is a 237kDa member of the *ferlin* family of transmembrane proteins involved in calcium-dependent sarcolemma repair and vesicle trafficking [reviewed in Cardenas et al. (2016) and Bulankina and Thoms (2020)]. Following physical injury to the sarcolemma, dysferlin facilitates the recruitment, and fusion of intracellular patches at the site of injury in an attempt to reseal damage sections of the cellular membrane (Bansal et al., 2003). Blunted dysferlin expression results in limb-girdle muscular dystrophy type 2B (LGMD2B) and Miyoshi myopathy, which leads to progressive muscle weakness in humans and rodents, and highlights an important role of dysferlin in skeletal muscle homeostasis (Harris et al., 2016; Straub et al., 2018).

Despite its critical role in sarcolemmal repair, positive dysferlin expression can be observed in non-skeletal muscle tissues and cell types. Indeed, blood monocytes and macrophages also express dysferlin (Nagaraju et al., 2008; Zhang et al., 2020), and its presence can also be found at the intercalated disks of cardiomyocytes (Chase et al., 2009), whereas our team has documented its presence in endothelial cells (Sharma et al., 2010), suggesting that dysferlin may regulate more than sarcolemmal repair events following physical injury. Conversely, very little is known about the presence of membrane repair mechanisms in non-muscle cells. In the case of endothelial cells however, their unique location between circulating blood and underlying tissue generates laminar sheer stress that may require membrane repair or remodeling. In addition, the harsh oxidative potential of blood and atherogenic lipids (Yang et al., 1999) may warrant a functional repair system, which could rationalize the well-documented low turnover rate of endothelial cells known to occur in normal vasculature (Woywodt et al., 2002). Besides modulating membrane remodeling and repair events, we have shown that dysferlin also regulates endothelial cell adhesion and angiogenesis, illustrating functional heterogeneity between endothelial ferlins (Bernatchez et al., 2007; Sharma et al., 2010). Whether dysferlin plays other roles in the vasculature in settings associated with chronic vascular injury and remodeling is unknown.

Atherosclerosis is a chronic disease that causes vascular remodeling and is associated with local inflammation and oxidative stress. Disruption of endothelial cell homeostasis is an early step in the atherosclerotic process (Ross, 1999; Yang et al., 1999), as it can lead to vascular permeability, impaired clotting function and lipid infiltration into the perivascular intima (Bonetti et al., 2003; Moore and Tabas, 2011; Wang et al., 2021), which likely causes stress injuries to the vascular wall. Despite the effects of dysferlin-deficiency on endothelial and vascular function, to date, atherosclerotic plaque development has not been explored in human patients or mouse models of dysferlinopathies. To test whether dysferlin protects the vasculature against atherosclerosis, we generated dysferlin-null mice on an apolipoprotein E-null (ApoE) background supplemented with a high fat diet (HFD) for 5 and 9 months (mo) to induce hyperlipidemia. Though we detected high dysferlin expression in rodent and human atherosclerotic lesions, we found that the loss of dysferlin does not affect plaque burden. We also report positive dysferlin expression in the liver and small intestine, the sites of lipoprotein metabolism regulation and lipid absorption, respectively, and plasma lipoprotein analyses revealed that loss of dysferlin modulates the plasma lipoprotein profiles of mice. We show that loss of dysferlin does not interfere with the efficacy of plasma lipid lowering mediated by cholesterol absorption blockade in mice lacking ApoE. Hence, dysferlinopathic patients are more likely to become dyslipidemic but remain responsive to cholesterol absorption blockade. This work further suggests that dysferlinopathies may have a metabolic or endocrine component.

## Materials and Methods

### Animal Models and Husbandry

All animals were housed in a 12-h/12-h light/dark cycle, temperature-regulated facility. All animal procedures were carried out in accordance with the guidelines and regulations set by the University of British Columbia (UBC), Animal Care Committee, and the UBC Animal Ethics Committee, Vancouver, BC, Canada. Experimental mice were generated by crossing B6.129-*Dysf*^TM^^1Kcam^ (Dysf) mice with the B6.129P2-*Apoe*^TM^^1Unc^/J (ApoE) mice to generate littermates heterozygous for both genes as previously described (Sellers et al., 2018). Littermates were bred together to produce WT, ApoE^–/–^, Dysf^–/–^ and Dysf^–/–^ApoE^–/–^ cohorts. Ear-clip DNA was extracted using the DNeasy extraction kit (Qiagen, #69506) following manufacturer’s instructions. Disruption of the Dysf gene was confirmed in mice via the original PCR protocol developed by and obtained directly from the Campbell laboratory (University of Iowa) using a long range PCR enzyme (Takara, #RR0002M) and the following primer sequences (common primer 5′-GCCAGACAAGCAAGGTTAGTGTGG-3′, wild-type primer 5′-GCGGGCTCTCAGGCACAGTATCGC-3′ and mutant primer 5′-CAGGGGCGCCCGGTTCTTTTTGTCAA-3′) (Han et al., 2011). ApoE mice were genotyped using the protocol suggested by Jackson using TopTaq DNA Polymerase (Qiagen; #200205) and the following primer sequences; (forward common 5′-GCCTAGCCGAGGGAGAGCCG-3′, wild-type reverse 5′-TGTGACTTGGGAGCTCTGCAGC-3′ and mutant reverse 5′-GCCGCCCCGACTGCATCT-3′).

Experimental mice were placed on a high fat diet (HFD) (Harlan, TD88137; 0.2% cholesterol, 21% total fat and 34% sucrose by weight) from 2 months of age until sacrifice following 5 and 9 months of HFD-feeding. Age-matched mice fed a regular chow diet (Chow; LabDiet #5001) served as controls for the 9 months time-point. Where applicable, ezetimibe (crushed pills) was activated in a 1:1 ratio of EtOH and added to drinking water for 9 months at a 15 mg/kg/d dose starting from 2 months of age. Dosage was titrated based on body weight and water consumption (averaged per cage). Ezetimibe is a novel sterol-absorption inhibitor that blocks NPC1L1-mediated cholesterol absorption of both dietary and biliary origin at the apical brush border membrane of enterocytes and thus lowers plasma lipoprotein concentration (Altmann et al., 2004). Following dietary and/or drug treatment, mice were sacrificed under terminal anesthesia (3.5% v/v isoflurane, 2L O_2_) via cardiac puncture and perfused with warmed Krebs solution [118 mmol/L NaCl, 22.5 mmol/L NaHCO_3_, 4 mmol/L KCl, 1.2 mmol/L NaH_2_PO_4_, 2 mmol/L CaCl_2_, 2 mmol/L MgCl_2_, and 11 mmol/L dextrose].

### Heart and Aorta Histology

Hearts and aortas were excised and fixed in 10% formalin. Following fixation, hearts were cut transversely 1 mm below, and parallel to, the atrioventricular plane. The aortic root was embedded and frozen in OCT, sectioned at 8 μm and stained with oil red O (Mehlem et al., 2013). Whole aortas were cleaned and stained with Sudan IV. Briefly, cleaned aortas were rinsed in 70% EtOH, placed into Sudan IV (5g Sudan IV in 500 mL 70% EtOH and 500 mL acetone) for 20 min, rinsed in two exchanges of 80% EtOH and then washed in running tap water for 60 min. Stained aortas were stored in 10% formalin until required. Photographs of pinned open aortas (opened en face) were taken for analysis, and percentage of plaque coverage was determined by dividing plaque area by total vessel wall area. Plaque was quantified in Aperio ImageScope and expressed as a percentage of total area.

### Analysis of Plasma Cholesterol and Lipids

Plasma was collected in heparinized tubes via cardiac puncture, spun down at 4,000RPM for 10 min at 4°C and stored at −80°C. The Siemans Advia 1800 system was used to quantify plasma concentrations of Cholesterol (CHOL-2), high density lipoprotein (D-HDL) and triglyceride (TRIG-2) levels (assays all from Siemans) and assays were performed according to instructions from the manufacturer and as previously published (Sellers et al., 2018).

### Cell Culture

Native bovine aortic endothelial cells (BAEC) were isolated from bovine aorta cells and grown in high glucose Dulbecco’s modified Eagle’s (DMEM; Gibco; #11965-084) supplemented with 5% FBS (Hyclone, South Logan, UT, United States) and 1% penicillin-streptomycin (Gibco; #15140-122). Human-derived hepatoma cells (HepG2) were grown in high glucose DMEM supplemented with 10% FBS and 1% penicillin-streptomycin as above.

### Western Blotting and Immunohistochemistry

Freshly dissected tissues (quadriceps, liver, thoracic aorta and small intestine) from WT and Dysf mice were excised, flash-frozen in liquid nitrogen and ground into a fine powder. Tissues were homogenized in a lysis buffer (pH 7.4) containing 50mM Tris-HCl; 1% NP40; 0.1% SDS; 0.1% Deoxycholic acid; 0.1mM EDTA and 0.1mM EGTA, supplemented with complete EDTA free protease inhibitor and PhosSTOP phosphatase inhibitor tablets (Roche, Manheim, Germany). Samples were further solubilized by sonication (4 × 5 s bursts at 40% amplitude; Fisher Scientific, Sonic Dismembrator Model 100), incubated on ice for 30 min and then centrifuged at 12,000RPM for 10 min at 4°C. BAEC and HEPG2 cells were lysed and solubilized as described above. Protein lysates were quantified using a standard Bradford assay. Samples were resolved on a 7.5% SDS-PAGE gel (Bio-Rad, Canada, #456-1024) and transferred onto nitrocellulose membranes (Bio-Rad, Canada, #170- 4158) using the Trans TurboBlot system set at 0.4A for 45 min (Bio-Rad, Canada). Following ponceau staining, membranes were blocked in 1% Casein in TBS (Bio-Rad, Canada, #1610782) and immunoblotting performed using the mouse anti-dysferlin antibody (Novocastra NCL Hamlet; Leica Biosystems; 1:100 dilution) or GAPDH (Cell Signaling Technology, # 2118; 1:5000) in a blocking buffer supplemented with 0.1% Tween20, overnight at 4°C. Membranes were incubated in either anti-mouse (Rockland; #610-145-003; 1:2500) or anti-rabbit (Thermo Fisher Scientific, #A-21038; 1:2500) secondary antibodies for 1–2 h at RT (in blocking buffer with 0.1% Tween20). Fluorescent signal was captured using the Odyssey CLx Imaging System (LICOR) and digital images were generated. Resultant images were converted into black and white using LICOR software.

Sections of human coronary artery specimens with diagnosed atheromatous lesions were obtained from the Heart and Lung Tissue Registry biobank at St. Paul’s Hospital as approved by the UBC Human Ethics Committee. For immunohistochemistry, paraffin tissues were probed with a goat anti-dysferlin antibody as previously described (Sharma et al., 2010) using citrate antigen retrieval.

### Statistical Analysis

Statistical analyses were performed using GraphPad Prism 6 and statistical analyses are described in figure legends. *P* < 0.05 was considered statistically significant. Figures show data as mean plus standard error of the mean (SEM) and N indicates the number of mice per experiment.

## Results

### Atheromatous Vessels of Mouse and Human Origin Show Robust Medial and Endothelial Dysferlin Expression

**FIGURE 1:**
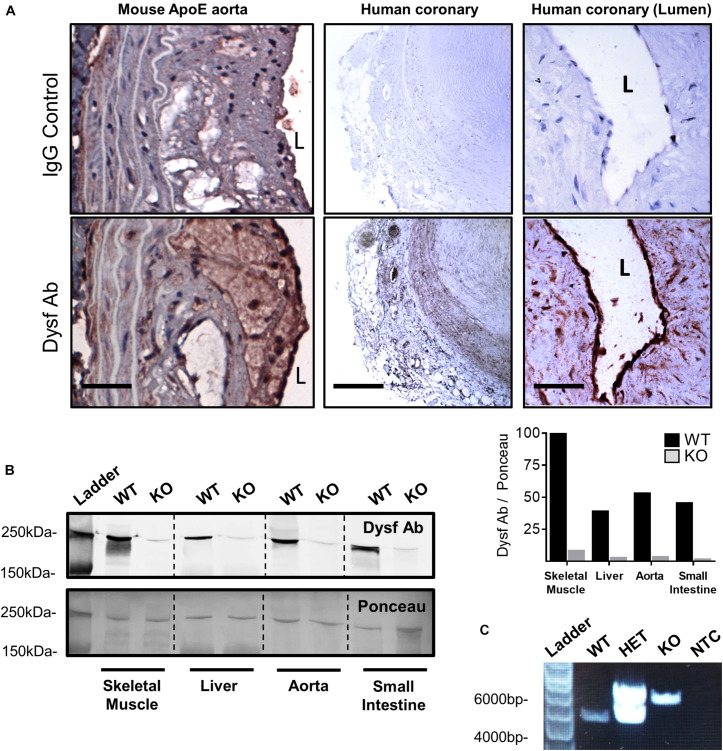
High dysferlin expression in mouse and human atherosclerotic lesions. **(A)** Dysferlin Ab and control detection in representative ApoE-null mouse aortic sections (scale bar = 40 μm) and human coronary lesions (scale bar = 600 μm and scale bar = 50 μm) using goat anti-dysferlin antibodies or IgG with hematoxylin counterstain. **(B)** Tissue specific detection and knockout efficiency of Dysferlin in mouse lysates (skeletal muscle, liver, thoracic aorta, and small intestine) via western blot using the mouse anti-dysferlin; NCL-Hamlet 1 antibody in wild type (WT) and Dysf (KO) mice. Quantification of Dysf Ab is shown relative to ponceau staining, and expressed as a percentage of the WT:skeletal muscle signal. **(C)** Genotyping PCR using ear clip DNA biopsies of wild type (WT), heterozygous mutant (HET), and homozygous mutant Dysf (KO) mice, as well as a no template control (NTC).

Having previously shown that dysferlin is highly expressed in healthy vessels (Sharma et al., 2010), we sought to determine its expression in atherosclerotic lesions. Dysferlin expression (brown) was detected in representative atherosclerotic lesions of ApoE-null mice, particularly in the intima and to a lesser extent the media, as well as in human atherosclerotic sections, with four and six-fold increases in staining observed when compared to control IgG-stained vessels, respectively (Figure 1A; *P* = 0.002 Fisher’s Exact Test, representative images 5 out of 5, or 100%, per group). In both mouse and human atheromatous lesions, particularly high dysferlin expression was observed by the lumen (L; Figure 1A), confirming high dysferlin expression in atherosclerotic and healthy endothelia (Sharma et al., 2010). Western blotting of protein extracts from WT mouse tissues also showed robust expression of dysferlin in control skeletal muscle lysates, as well as in the liver, aorta and small intestine, whereas dysferlin-null tissues showed near-complete loss of dysferlin expression (Figure 1B).

### Loss of Dysferlin Does Not Affect Aortic Atherosclerosis Development

**FIGURE 2:**
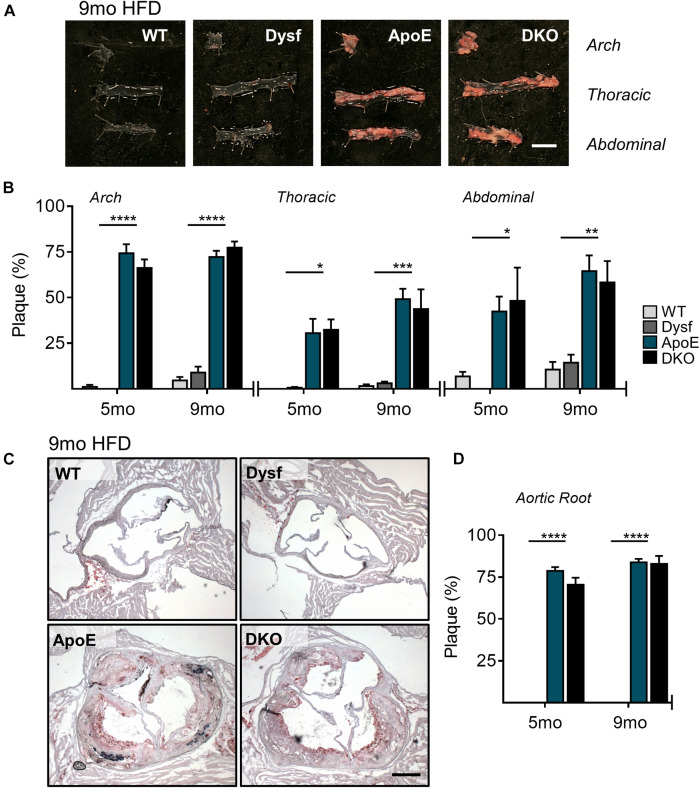
Similar atherosclerotic plaque accumulation in aortic segments from ApoE and DKO mice after 5 and 9 months (mo) of HFD-feeding. **(A)** Representative images of Sudan IV stained plaque accumulation in distal, arch, thoracic, and abdominal aortic segments from WT, Dysf, ApoE, and DKO mice after 9 months of HFD-feeding, and quantification of plaque accumulation after both 5 and 9 months of HFD-feeding **(B)**, 5 months: WT (*N* = 5), Dysf (*N* = 5), ApoE (*N* = 6), and DKO (*N* = 4), 9 months: WT (*N* = 6), Dysf (*N* = 6), ApoE (*N* = 5), and DKO (*N* = 7). Mean + SEM. WT and Dysf genotypes significantly different from ApoE and DKO at each time-point; *P* < 0.05 (*); *P* < 0.01 (**); *P* < 0.001 (***); *P* < 0.0001 (****); two-way ANOVA with Sidak’s *post hoc* tests. No significance changes to atherosclerosis was detected between 5 and 9 months-fed cohorts; two way ANOVA. Scale bar = 0.5 cm. Similar atherosclerotic plaque accumulation in the aortic sinus of ApoE and DKO mice after 5 and 9 months (mo) of HFD-feeding. **(C)** Representative images of Oil Red O stained plaque accumulation in the aortic root sinus of WT, Dysf, ApoE, and DKO mice after 9 months of HFD-feeding, and quantification of plaque accumulation after both 5 and 9 months of HFD-feeding **(D)**, 5 months: WT (*N* = 7), Dysf (*N* = 4), ApoE (*N* = 6), and DKO (*N* = 4), 9 months: WT (*N* = 4), Dysf (*N* = 5), ApoE (*N* = 5), and DKO (*N* = 6). Mean + SEM. WT and Dysf genotypes significantly different from ApoE and DKO; *P* < 0.0001 (****). Two-way ANOVA with Sidak’s *post hoc* tests. No significance changes to atherosclerosis was detected between 5 and 9 months-fed cohorts; two way ANOVA. Scale bar = 0.5 cm.

Due to high dysferlin expression in atherosclerotic and aortic tissues (Figures 1A,B) and its role in endothelial adhesion during angiogenic stimulation (Sharma et al., 2010), normolipidemic and dyslipidemic ApoE-null mice – representing very slow and accelerated models of atherosclerosis, respectively, were bred to WT and dysferlin KO animals. Efficient deletion of dysferlin was confirmed in KO samples by both western blot, as well as by PCR (Figures 1B,C). After 5 months of HFD-feeding, little to no plaque was observed in aortic arch, thoracic and abdominal segments of WT mice, whereas ample atheromatous lesions were detected in ApoE-null animals using Sudan IV staining (Figures 2A,B). Loss of dysferlin did not result in significant changes to plaque burden in either normolipidemic or dyslipidemic models. After 9 months of HFD-feeding, basal plaque accumulation in normolipidemic mice did not exceed 18% in any segment, whereas in ApoE-deleted groups, atherosclerosis covered ≥ 43% of aortic surface area in each segment (Figures 2A,B). In all cases, loss of dysferlin did not significantly affect basal or dyslipidemia-induced atherosclerosis, which suggests that dysferlin does not protect against atherosclerosis in distal sections of the aorta.

To further document the role of dysferlin in atherosclerosis, we examined plaque burden in the aortic sinus of mice after both 5 and 9 months of HFD. Compared to ApoE-expressing groups, which were devoid of plaque, significant but similar increases in lipid deposition were observed in ApoE and DKO aortic root sections, covering 78 and 84% of the total area after 5 and 9 months of HFD, respectively (Figures 2C,D). Hence, in multiple sections of the aorta, the loss of dysferlin did not impact atherosclerosis development.

### Loss of Dysferlin Alters Plasma Lipoprotein Composition in an ApoE-Sensitive Manner

**FIGURE 3:**
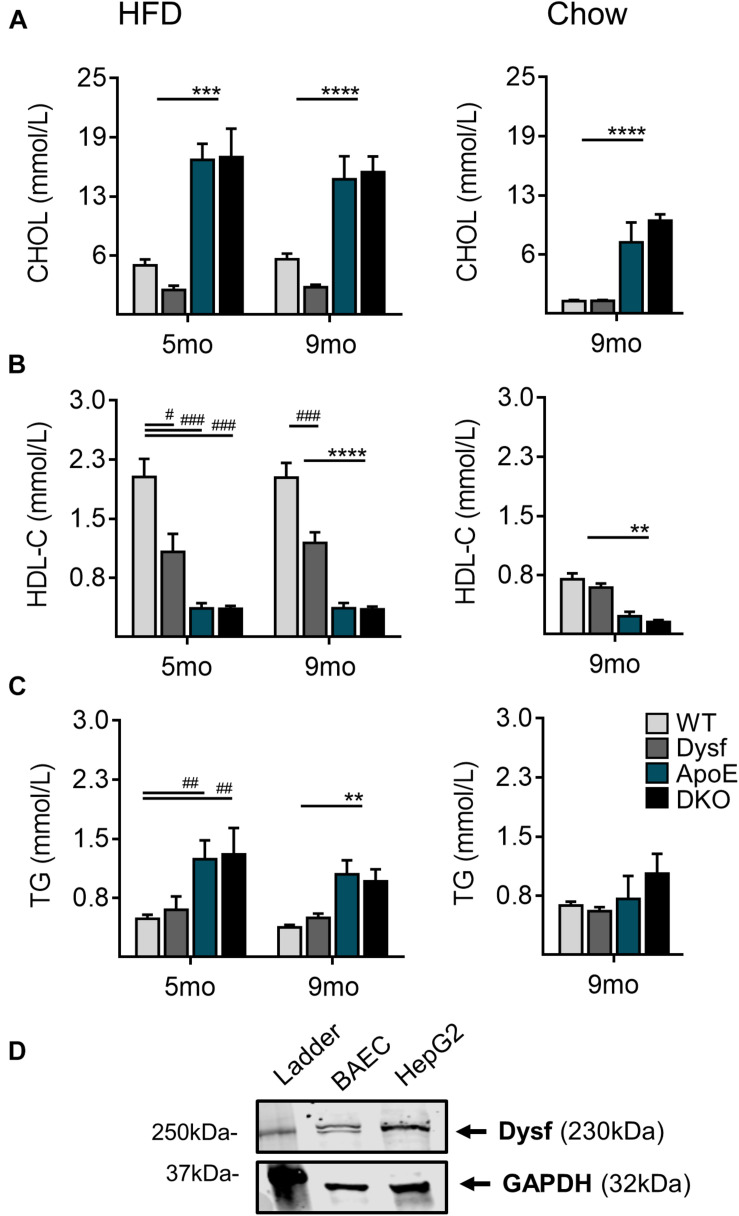
Total plasma cholesterol, high density lipoprotein cholesterol and triglyceride levels in WT, Dysf, ApoE and DKO mice after 5 and 9 months (mo) of HFD-feeding and age-matched chow controls at 9 months. **(A)** Total cholesterol (CHOL) **(B)**, high density lipoprotein (HDL-C), and **(C)**, Triglycerides (TG). **(D)** Protein extracts from dysferlin-expressing BAEC (control cell lysate), and a its expression in a human hepatocyte derived cell line (HepG2). GAPDH is used as a loading control. 5 months HFD: WT (*N* = 10), Dysf (*N* = 5), ApoE (*N* = 7), DKO (*N* = 5), 9 months HFD: WT (*N* = 13), Dysf (*N* = 14), ApoE (*N* = 11), and DKO (*N* = 11). WT and Dysf genotypes significantly different from ApoE and DKO; *P* < 0.01 (**); *P* < 0.001 (***); *P* < 0.0001 (****); Significantly different from WT; *P* < 0.05 (^#^); *P* < 0.01 (^##^); *P* < 0.001 (^###^); two way ANOVA with Sidak’s *post hoc* tests, 9 months Chow: WT (*N* = 8), Dysf (*N* = 11), ApoE (*N* = 4), AND DKO (*N* = 7). WT and Dysf genotypes significantly different from ApoE and DKO; *P* < 0.01 (**); *P* < 0.0001 (****); One way ANOVA Tukey’s *post hoc* tests. Mean + SEM.

Consistent with increased plaque accumulation, ApoE, and DKO mice displayed similar elevations in total plasma cholesterol (CHOL), as well as corresponding decreases in athero-protective plasma HDL-C concentrations after both 5 and 9 months of HFD-feeding (Figures 3A,B). A 50% and 51% decrease in plasma CHOL, and circulating HDL-C (47% and 41%; *P* < 0.0001) was also observed in Dysf-null compared to WT mice at both time-points (Figures 3A,B), whereas circulating triglycerides (TG) were similar between normolipidemic and dyslipidemic Dysf-null mice and their appropriate controls (Figure 3C). Similar patterns of plasma lipoprotein composition were observed in age-matched ApoE and DKO mice fed a regular chow diet for 9 months, whereas under normolipidemic conditions WT and Dysf-null mice display similar plasma lipid profiles. Since western blotting revealed abundant dysferlin protein expression in WT, but not Dysf-null liver extracts (Figure 1B), and dysferlin gene deletion results in changes to plasma CHOL and HDL-C, dysferlin may therefore play a role in plasma lipoprotein metabolism. As the liver contains many cell types, including dysferlin-expressing endothelial cells, dysferlin protein expression was also confirmed in a human-hepatocyte-derived cell line (HepG2) (Figure 3D).

### Loss of Dysferlin Does Not Interfere With Ezetimibe-Induced Cholesterol Absorption Blockade

**FIGURE 4:**
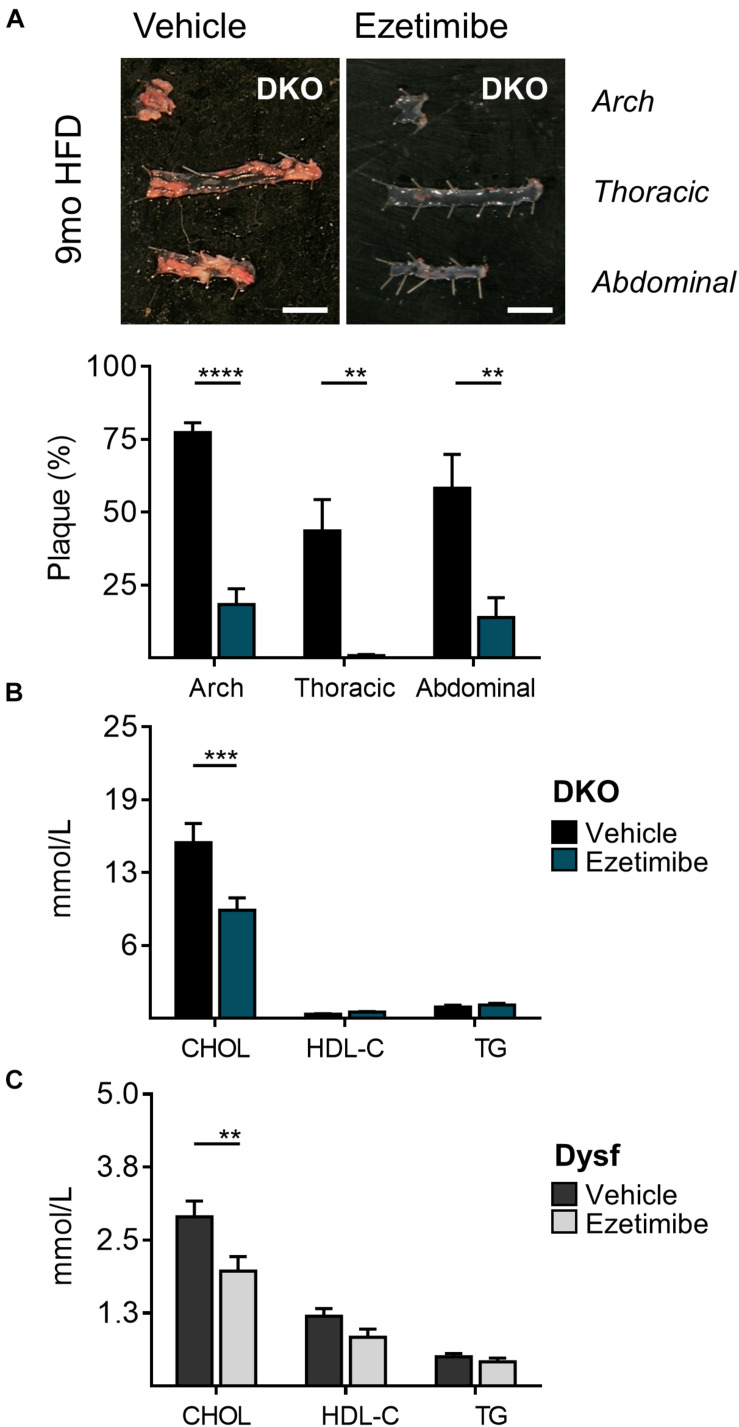
Decreased atherosclerosis and plasma cholesterol levels in DKO mice treated with the cholesterol absorption blocker ezetimibe for 9 months. **(A)** Representative images and quantification of plaque accumulation from aortic arch, thoracic and abdominal segments, **(B)** total plasma cholesterol (CHOL), high density lipoprotein (HDL-C), and triglyceride (TG) levels from HFD-fed DKO mice, and **(C)** CHOL, HDL-C, and TG levels from HFD-fed Dysf-null mice treated with vehicle or ezetimibe for 9 months. DKO: Vehicle (*N* = 11), Ezetimibe (*N* = 7); Dysf: Vehicle (*N* = 14), Ezetimibe (*N* = 9); Significance at *P* < 0.01 (**); *P* < 0.001 (***); *P* < 0.0001 (****); Two way ANOVA Sidak’s *post hoc* tests. Mean + SEM. Scale bar = 0.5 cm.

Dyslipidemia often dictates the use of cholesterol-lowering medications. Since dysferlinopathic patients have weakened muscles, we tested the possibility of lowering atherogenic cholesterol with ezetimibe, an intestinal cholesterol absorption blocker, which compared to statins, does not require functional ApoE expression to lower atherogenic plasma cholesterol (Bea et al., 2002; Altmann et al., 2004; Nachtigal et al., 2006; Tie et al., 2015) and may be preferred in settings of rhabdomyolysis (Perez-Calvo et al., 2005). Since abundant dysferlin expression was observed in protein extracts from the small intestine of WT mice (Figure 1B), Dysf-null and DKO mice were treated with ezetimibe. In dyslipidemic DKO mice, treatment with ezetimibe resulted in reduced plaque burden in aortic arch, thoracic and abdominal segments by 71, 93, and 75%, respectively, compared to vehicle treated mice after 9 months of HFD-feeding (Figure 4A). In addition, plasma CHOL, but not HDL-C or TG levels, were reduced by 38% in DKO mice, respectively (Figure 4B). A comparable 32% decrease in plasma CHOL levels were also observed in HFD-fed Dysf-null mice treated with ezetimibe compared to vehicle controls in absence of profound atherosclerosis, further confirming that dysferlin does not effect intestinal cholesterol blockade, despite robust enterocyte expression (Figure 4C).

## Discussion

The data presented herein suggest that dysferlin, a physical repair protein, is highly expressed by atheromatous medial and intimal cells. Since we hypothesized that dysferlin may participate in the repair and maintenance of vascular cells, we generated normal and dysferlin-deficient mice with or without expression of ApoE. These mice were fed a high fat diet to increase atherogenic plasma lipids, and we unexpectedly observed that loss of dysferlin does not increase basal or hyperlipidemia-accelerated atherosclerosis. We do report that dysferlin likely regulates plasma lipoprotein homeostasis and that it is highly expressed by the liver and small intestine, which are key organs for lipoprotein metabolism and lipid absorption. Despite these abnormalities, loss of dysferlin does not interfere with the cholesterol absorption blocking and anti-atherosclerotic properties of ezetimibe. This could have implications in dysferlinopathic patient management, as they may present with dyslipidemia and remain responsive to ezetimibe, without necessarily being at greater atherosclerotic risk.

We have recently demonstrated that loss of dystrophin, another sarcolemmal rigidity protein that causes Duchenne or Becker MD when mutated, results in a new type of primary genetic dyslipidemia both in patients and unmedicated mutant animals (White et al., 2020). When taken together with the data presented herein, human dysferlinopathies, which result in LGMD2B and MM, could be another type of MD with characteristic dyslipidemia (White et al., 2020). Indeed, early high-resolution NMR analysis of DMD patient serum and human LGMD2B muscles showed a significant increase in total cholesterol, free cholesterol, cholesterol esters, triglycerides and certain phospholipids compared to unaffected age- and gender-matched controls (Srivastava et al., 2010, 2017), although the lipoprotein data in the current study was obtained with routine clinical tools. While we confirmed dysferlin expression in mouse liver samples, how loss of dysferlin may alter lipoprotein metabolism is unknown. A candidate is the dysferlin-dependent expression of VLDLR, a key lipoprotein regulator which is down-regulated in dysferlin-null mice (Suzuki et al., 2005). Whether a change in VLDLR is the underlying cause of the lipid profile abnormalities we describe in the current study is unknown but VLDLR is highly homologous to LDLR and binds ApoE- but not ApoB-containing lipoproteins, the former being most present on non-HDL-C particles. Despite these deficiencies, loss of dysferlin does not interfere with the blockade of cholesterol absorption, which is typically mediated by the intestinal Nieman-Pick C1-like 1 transporter and could be of clinical relevance since dysferlinopathic patients display similar plasma lipid abnormalities as Duchenne MD individuals (White et al., 2020). To err on the side of caution, one must note the major differences between the lipid profiles of WT and ApoE-null mice and that of humans. WT and ApoE –null mice typically carry most of their cholesterol through HDL and LDL particles, respectively, whereas human plasma typically is LDL-rich. Hence, these findings should be confirmed in dysferlinopathic patients.

Atherosclerosis is a complex process that involves multiple cells types that incidentally express dysferlin. Indeed, endothelial cells, smooth muscle cells, and monocytes (De Luna et al., 2007; Gallardo et al., 2011), the precursor of macrophage-derived foam cells, all express dysferlin. In endothelial cells, dysferlin was shown to play an important role in angiogenic signaling and cell adhesion under active growth conditions (Sharma et al., 2010), whereas dysferlin expression in monocytes regulates the immune response (Chiu et al., 2009), inflammatory marker upregulation (Rawat et al., 2010), adhesion molecule trafficking (de Morrée et al., 2013), and increased cell motility and phagocytic activity (Nagaraju et al., 2008). In contrast, the role of dysferlin in smooth muscle cell biology is completely unknown. Hence, one might suggest that our full knock out approach may mask a more complex and heterogeneous role for dysferlin that could only be dissected using cell-specific KO approaches. Another limitation is plaque composition and volume, which might be dysferlin-dependent but was not investigated. Potential compensatory mechanisms from other ferlins, like endothelial myoferlin also remain a possibility, although we have shown that ferlins are heterogeneous in their vascular biological properties (Bernatchez et al., 2007; Sharma et al., 2010; Yu et al., 2011). In contrast, recent studies investigating the role of dystrophin, another sarcolemmal integrity protein, have demonstrated its anti-atherogenic properties (Shami et al., 2015; Milad et al., 2017), amongst other vascular reactivity properties (Hoffman et al., 1988; Loufrani et al., 2001), with reduced atherosclerotic plaque load in dystrophin-deficient ApoE-null mice, and a lower number of inflammatory and smooth muscle cell infiltrates into the aortic lesion, which suggests that dystrophin may play a role in modulating the immune contribution to atherogenesis (Shami et al., 2015). Thus, while both dystrophin and dysferlin are expressed in the endothelium and are associated with the development of muscular dystrophies, they play vastly different roles in vascular homeostasis and indeed the progression of vascular disease.

## Data Availability Statement

The raw data supporting the conclusions of this article will be made available by the authors, without undue reservation.

## Ethics Statement

The animal study was reviewed and approved by UBC Animal Care Committee.

## Author Contributions

PB, SS, NM, and ZW contributed to conception and design of research. ZW, NM, and SS performed experiments and interpreted results. ZW and NM analyzed data and prepared figures. PB, ZW, and NM wrote the manuscript. All authors approved final version of the manuscript.

## Conflict of Interest

The authors declare that the research was conducted in the absence of any commercial or financial relationships that could be construed as a potential conflict of interest.
